# Professional Benevolence

**Published:** 1910-06-18

**Authors:** 


### The Hospital

A Journal of

The Medical Sciences and Hospital Administration.

New Series. No. 175, Vol. VII. [No. 1247, Vol. XLVIII.] SATURDAY, JUNE 18 1910

### PROFESSIONAL BENEVOLENCE.

The medical profession does not stand conspicuously above the sister professions in providing for its members an assured recompense for the work that they have to do. It differs from all other professions in that it exacts from those who belong to it greater self-sacrifice, a larger amount of unremunerative work, and a greater willingness to face certain dangers which, although medical men would be the last persons in the world to harp upon them, are nevertheless constant and ever present, and which all of us have to take into account. We are addressing fellow-practitioners, not the lay public, and there is no necessity to lay stress on the platform examples which those who in one moment laud the merits of doctors to the skies and in the other forget the benefits which the presence of skilled medical assistance affords to the community, are always ready to bring forward when it suits their convenience to do so. Every medical man can recall many instances of heroism and devotion to duty among his colleagues of which the outside public knows nothing. It is a trite truism that the struggle for existence is becoming more strenuous every day in all departments of human activity, and it is a well-known fact that medical men are not exempt from the disadvantages that such competition entails. When we bear in mind that the average income of doctors in these islands is well below £120 a year, and take into consideration the additional fact that this income has to be earned in a manner which, through its worry and stress, is not conducive to longevity, it is at once apparent that the need for helping those who have, through no fault of their own, fallen behind in the struggle is very great. Can it be wondered that many medical men find it impossible adequately to provide for their families, or for their own future when they are broken and incapacitated, during a career which is so strenuous and, judging by that average income, so unremunerative? It is true there are the fortunate few whose "living wage" is far above that of their less lucky colleagues, and whose careers have been singularly blessed. To those the appeal that the Medical Benevolent Fund Guild makes on behalf of the less fortunate should prove a powerful call to action. What the objects of that Guild are has already been shown in the addresses delivered on the occasion of the Guild's special meeting which we chronicled in our issue of June 4 last. Briefly put, it is an organisation that endeavours to help and assist the families of poor medical men whose means are insufficient to ensure them comparative comfort. With that object every member of the profession ought to be in cordial sympathy. Everyone who has a sense of colleagueship and brotherliness should attempt to do his utmost to support the Guild in the good work it is doing. The Guild needs the help of the successful practitioner, the man who can spare from his income, large in comparison with the meagre earnings of the average, a mite towards the funds of the Guild. But it needs more. It needs the help of every practitioner, no matter whether he is rich or poor. Those who cannot themselves give can at least advertise to the best of their abilities the benevolent object of the Guild. True enough, the Fund appeals primarily to the profession, but the public as well must be made to realise that it has a legitimate claim to share in the work.

There are few more deserving or more beneficial charities that can interest the practitioner than this, and we sincerely trust that the Guild will make itself known as widely as possible. Many members of the profession, we feel sure, do not even know of its existence; many certainly have no conception of the good it has accomplished and that it may yet accomplish. A personal appeal, by means of a circular letter, setting forth the aims and objects of the Guild, would surely not be without success in awakening the mass of the profession to a sense of the obligations it owes to those who have fallen behind, and we would urge upon the executive of the Guild the desirability of making such an appeal. The example set by Sir Alfred Fripp is one that is well worthy of imitation by other leaders in the profession. If prominent surgeons and physicians would actively associate themselves with the movement, if they would bring it to the attention of their students and fellow-practitioners, and by example and precept show that they are fully alive to the importance of the work that the Guild is doing, there would be no reason to fear that want of funds will in the future hamper or limit the benevolent intentions of the association.

